# Effect of different load of shoulder external rotation exercises on changes in muscle activity and exerted torque

**DOI:** 10.3389/fspor.2025.1527296

**Published:** 2025-02-18

**Authors:** Yurika Saeki, Atsushi Kubota, Kohei Kishimoto, Mika Inoue, Takumi Inoue, Yuji Takazawa

**Affiliations:** ^1^ Graduate School of Health and Sports Science, Juntendo University, Inzai, Japan; ^2^ College of Sport and Health Science, Ritsumeikan University, Kusatsu, Shiga, Japan; ^3^ Juntendo Administration for Sports, Health and Medical Sciences, Juntendo University, Hongo, Tokyo, Japan; ^4^ Department of Sports Medicine and Sportology, Graduate School of Medicine, Juntendo University, Tokyo, Japan

**Keywords:** rotator cuff, muscle activity ratio, shoulder conditioning, sports & exercise medicine, external rotation torque

## Abstract

The effects of shoulder external rotation exercises on the EMG amplitude of the infraspinatus, and teres minor, and torque in healthy individuals remain uncertain. In this study, we aimed to determine the effects of varying loads during shoulder external rotation exercises on exerted torque and muscle activity of the infraspinatus, teres minor, and deltoid. Twenty-four upper limbs from 12 healthy adult males (22.5 ± 1.9 years) were included. Participants performed shoulder external rotation exercises with low-, medium-, and high-load conditions using elastic bands of three different tensions. The number of exercises was set so that the total workload during the exercise was equal for each loading condition. The torque of the shoulder external rotation and electromyography (EMG) amplitude of the infraspinatus, teres minor, and the posterior deltoid were measured during the concentric shoulder external rotation task, before and after the exercise. In addition, the muscle activity ratio of the three muscles was calculated. Analysis divided into 30° intervals, under the low-load condition, shoulder external rotation torque and EMG amplitude of the infraspinatus and teres minor did not change; However, the EMG amplitude of the posterior deltoid increased significantly. The muscle activity ratio in the posterior deltoid showed exercise range × time interaction, with a significant increase from pre-exercise (Pre) (13.59 ± 5.70%) to 20 min after the exercise (15.40 ± 6.03%) in the 61°–90° external rotation range. In the medium- and high-load conditions, the EMG amplitude significantly increased for all muscles. However, under the medium-load condition, significant differences were observed between 0–30° (Pre: 25.4 Nm, 20 min: 26.0 Nm), 31–60° (Pre: 24.3 Nm, 20 min: 25.4 Nm), and 61–90° (Pre: 23.7 Nm, 20 min: 24.6 Nm). There was also an increase in the muscle activity ratio in the posterior deltoid, with a main effect on time in the medium load condition (*p* < 0.05). The changes in torque, EMG amplitude, and muscle activity ratio after the shoulder external rotation exercises were not uniform across different exercise loads. Therefore, it is necessary to use different tensions depending on the purpose of the exercise.

## 1 Introduction

The infraspinatus and teres minor, parts of rotator cuff muscles, help to dynamically stabilize the glenohumeral joint and to generate shoulder external rotation torque during shoulder movement. The low shoulder external rotation torque and the low external/internal rotation torque ratio are the risk factors for shoulder joint disorders and maintaining their function is essential for prevention of injuries (1, 2). Especially in overhead athletes, stronger contractions of the shoulder external rotators are required to keep the humeral head in an afferent position for rapid swinging of the upper extremity during pitching. The high electromyography (EMG) amplitude was reported during pitching motion, with approximately 74% and 84% maximum voluntary isometric contraction (MVIC) in the infraspinatus and teres minor, respectively (3). Therefore, incorporating shoulder external rotation exercises to promote muscle activity in the shoulder external rotator muscles and increase shoulder external rotation torque is important to prevent shoulder joint injuries.

The infraspinatus, teres minor and superficial posterior deltoid work together in a coordinated manner during shoulder external rotation, but the function of each muscle is different due to the varying orientation of their muscle fibers and attachments. The infraspinatus and teres minor rest on the greater tuberosity of the humerus and prevent the humeral head from leaving the afferent position of the glenohumeral fossa during shoulder joint motion. The posterior deltoid is located superficially around the shoulder joint, has a large muscle cross-sectional area and produces a shearing force on the humeral head. Therefore, if it has a greater muscle activity relative to the infraspinatus and teres minor, this may lead to a risk of shoulder impingement (4, 5). Hence, it is important to focus on the muscle activity ratio of these muscles to maintain the glenoid fossa and humeral head in an afferent position during shoulder motion.

Yu et al. reported that the muscle activity ratio of the infraspinatus to the posterior deltoid during exercise was smaller when the exercise load was higher (6). In contrast, Park et al. showed no difference in the infraspinatus/posterior deltoid muscle activation ratio when the exercise load was increased (7). Thus, there is no consensus on the effect of different exercise loads on the activity ratio of the infraspinatus and posterior deltoid. Exercises targeting the rotator cuff are often performed with low loads using elastic bands or light dumbbells to reduce a shearing force of the deltoid while promoting the rotator cuff's contribution (8, 9). However, the effects of shoulder external rotation exercises on the EMG amplitude and torque of the shoulder external rotation muscles remain unclear and there is little evidence to determine the load of the exercises. It is necessary to investigate the effects of different exercise loads on torque, EMG amplitude, and muscle activity ratio. It is then important to consider adjusting the load to suit the purpose of the exercise.

Hence, we aimed to determine the effects of shoulder external rotation exercises at different loads (low-, medium-, and high-load) on changes in external rotation torque, the EMG amplitude, and muscle activity ratio of the infraspinatus, teres minor, and deltoid. We hypothesized that increasing the load of shoulder external rotation exercises would increase shoulder external rotation torque and EMG amplitude without altering the muscle activity ratio of the shoulder external rotators after the exercise.

## 2 Materials and methods

### 2.1 Participants

We examined twenty-four upper limbs of 12 healthy adult males (age: 22.5 ± 1.9 years, height: 172.3 ± 4.8 cm, weight: 65.6 ± 9.2 kg) included in this study. The participants had not engaged in strength training or other strenuous exercise continuously for the past 1 year. Furthermore, they were fully informed about the experimental procedures, possible risks, and the purpose of this study. Exclusion criteria included individuals with a history of neuromuscular disease or disorder, musculoskeletal trauma or disorder to the shoulder joint within the past 6 months, a history of shoulder joint surgery, and hypersensitivity or allergy for skin treatment. Written informed consents were obtained from all participants before the experiment, and the study was approved by the Juntendo University Faculty and Graduate School of Sports and Health Science and the Research Ethics Committee (No. 2023-71).

The sample size was determined through a power analysis to ensure adequate statistical power. Based on an observed effect size (*f* = 0.38), an alpha level of 0.05, and a desired power of 0.80, the power analysis indicated that a sample size of 12 participants would be sufficient to detect meaningful effects. This sample size exceeds the required threshold for achieving the desired power, suggesting that the study is adequately powered.

**Figure 1:**
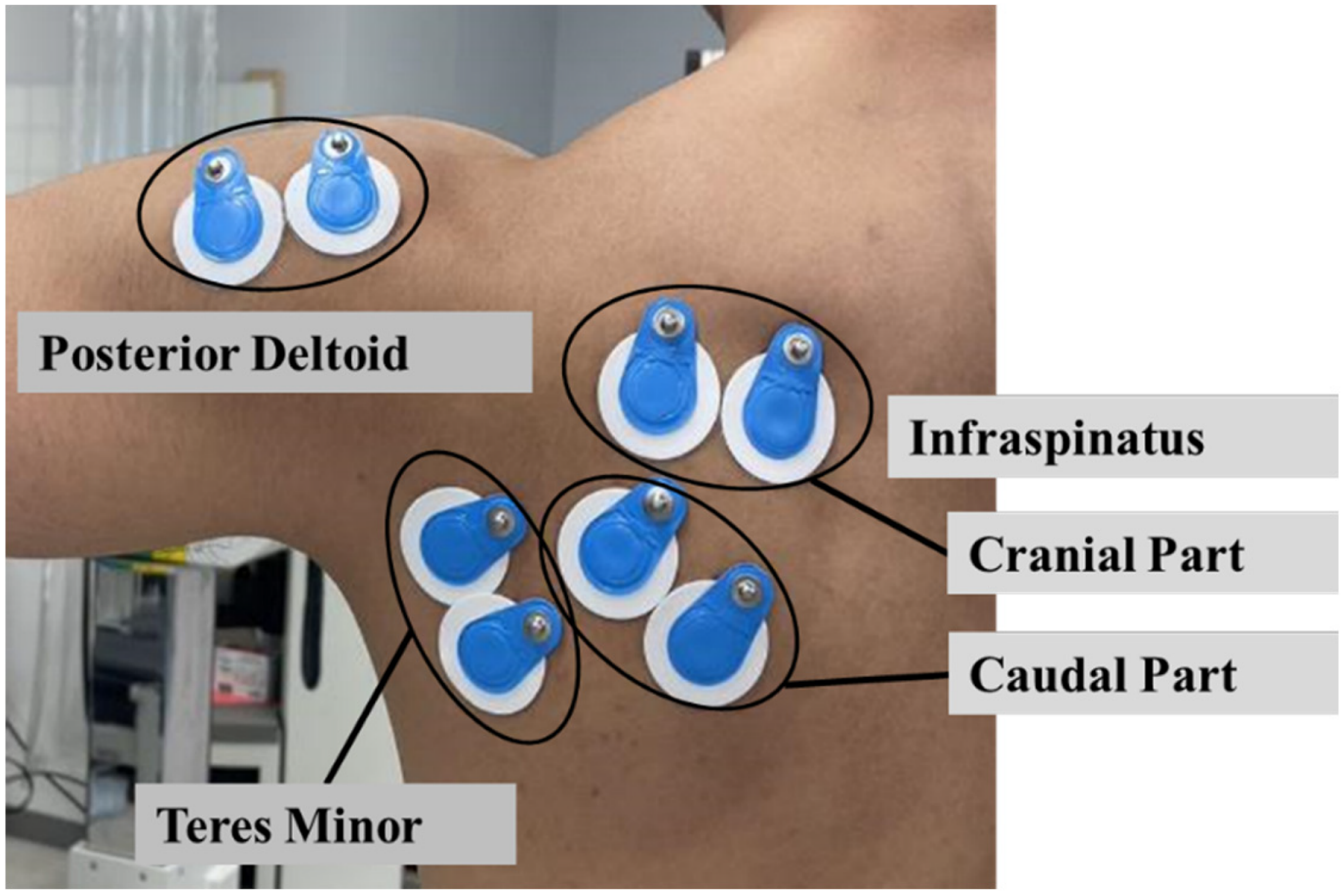
Electrodes on the infraspinatus, teres minor, and posterior deltoid.

To acquire electromyography (EMG) signals, a wireless EMG system, Ultium EMG (Noraxon, Scottsdale, Arizona, USA), was synchronized with the Biodex System 4. The EMG signals were sampled at 2000 Hz and bandpass filtered at 20–500 Hz to remove motion artifacts. Disposable electrodes (diameter 34 mm, Blue Sensor, M-00-S) were affixed at a distance of 35 mm between electrodes. The measurement areas were wiped with alcohol prior to electrode application to minimize skin impedance. Electrodes were placed while the shoulder joint was held in 90° abduction to align with the exercise position. The EMG signals were recorded at two sites, the cranial part and the caudal part, located in the superficial layer because the infraspinatus is structurally divided into three parts (10). For the cranial part of the infraspinatus, electrodes were placed 3–4 cm below the scapular spine (7, 11, 12) on the line connecting the posterior angle of the acromion and the midpoint of the medial edge of the scapular spine with the inferior angle. For the caudal part, they were positioned 1/3 of the way down the line connecting the medial edge of the scapular spine and the inferior angle, along the muscle fibers. Electrodes for the teres minor were placed in the upper 1/3 of the line connecting the posterior angle of the acromion to the inferior angle (13, 14), and for the posterior deltoid, they were positioned 2 cm below the posterior angle of the acromion along the muscle fibers (7, 12, 15). The electrodes were applied while the shoulder joint was held in 90° abduction aligning with the exercise position (Figure 1).

### 2.2 Procedure

**Figure 2:**
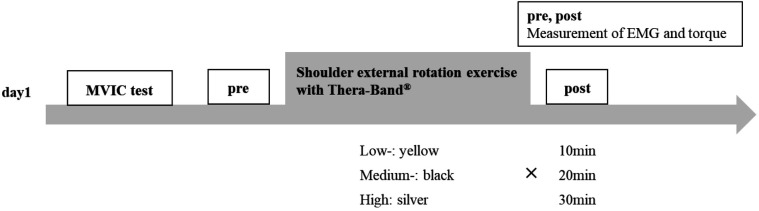
Procedure. Low-, medium-, and high-load 10 min, 20 min, and 30 min measurements were performed on separate days. A random assignment table was used to determine the order. MVC, maximum voluntary isometric contraction.

We used a crossover design in this study. First, an MVIC test of shoulder external rotation was performed. Subsequently, shoulder external rotation torque during shoulder external rotation was recorded before and 10, 20, and 30 min after exercises in each exercise condition. The measurements were taken during concentric contraction at an angular velocity of 60°/sec and EMG amplitude of the cranial part and caudal part of the infraspinatus, teres minor, and posterior deltoid. Furthermore, measurements at 10, 20, and 30 min were taken on separate days to avoid the influence of the measurement on the results (Figure 2). In addition, we used a random assignment table to determine the order in which all nine sessions were performed, ensuring that neither the measurement of shoulder external rotation torque nor the familiarity with exercises using the Thera-Band® affected the results. In addition, the EMG amplitude of each muscle was recorded during the exercises to ascertain differences in muscle activity during the exercises due to tension.

### 2.3 Maximum voluntary isometric contraction test

We performed an MVIC test of the shoulder external rotation before each session to normalize the EMG amplitude data and calculate the intensity during the exercises. The test was performed for 5 sec using the Biodex System4 (Biodex Medical Systems, Shirley, NY, USA) while participants were seated with the shoulder joint in 90° abduction, intermediate internal and external rotation, and elbow in 90° flexion. It was performed after practice with verbal encouragement and visual feedback of torque curves to demonstrate maximal isometric muscle strength. The intraclass correlation coefficient (1,1) in the shoulder external rotation torque during this test was 0.921 (95% confidence interval: 0.853–0.962).

### 2.4 Shoulder external rotation exercise

**Figure 3:**
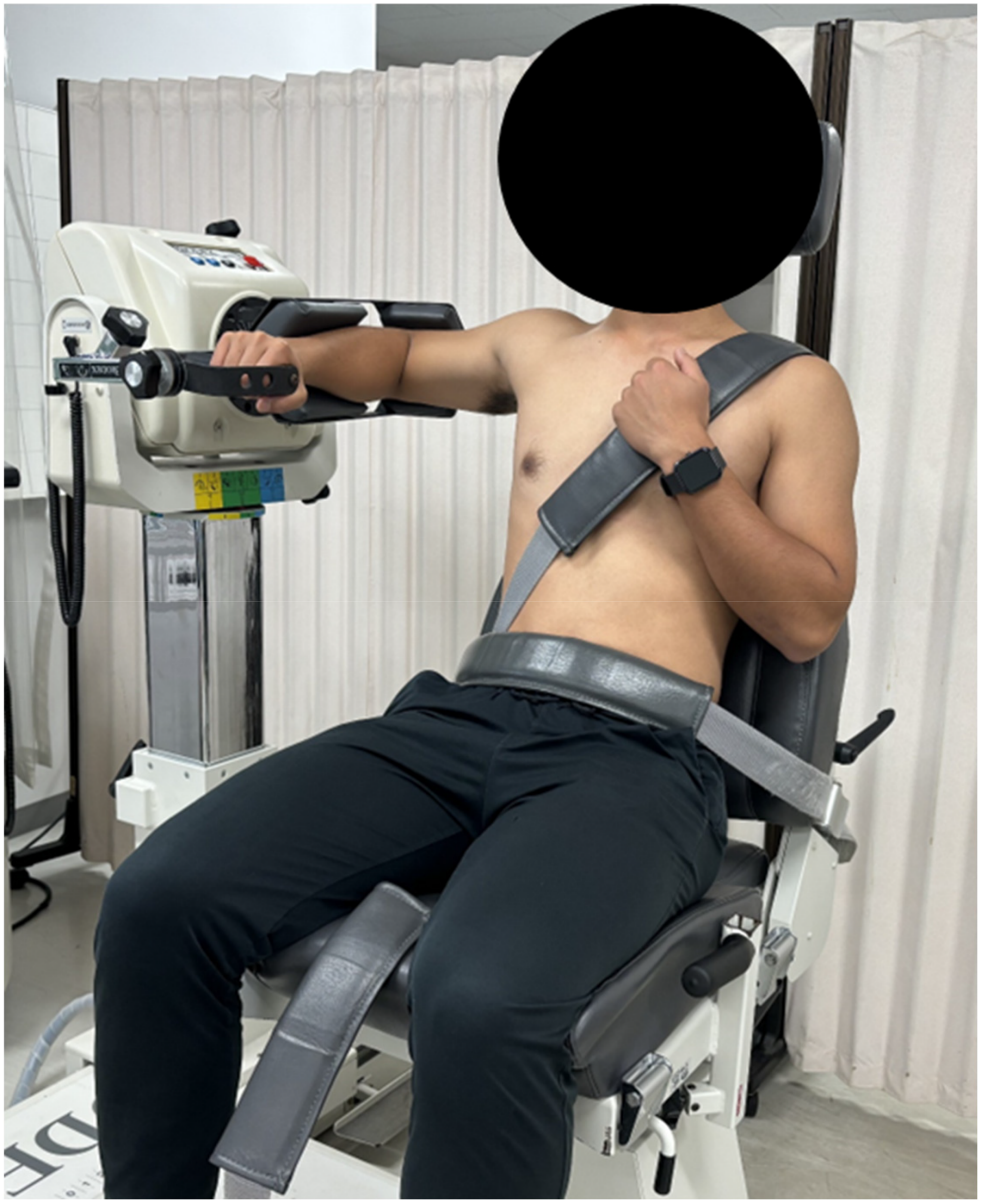
Shoulder external rotation exercise. External rotation exercises were carried out with the shoulder at 90 of abduction and the elbow at 90 of flexion. The participant sat on the dynamometer with the trunk fixed by a cross seatbelt.

Exercises were performed using Thera-Band® (Thera-Band® Hygenic Corporation, Akron, OH, USA) in yellow, black, and silver with different tensions for low-, medium-, and high-load conditions, respectively. Furthermore, the shoulder's external rotation exercises were performed in the 90° shoulder abducted position, which is similar to the overhead movement and is considered by many competitors to be incorporated as an exercise during warm-up. The upper body was immobilized with two attached belts in a seated position on the Biodex System 4 isokinetic muscle testing device (Figure 3) to reduce the compensatory effect of thoracic extension during the exercise. The participant was asked to grasp a 50 cm Thera-Band® extended to 1 m in a position with the shoulder abducted at 90°, the arm intermediate (midway between internal and external rotation), and the elbow flexed at 90°. The range of motion and velocity were equivalent to that of shoulder external rotation under concentric contraction before and after the exercise, the participants performed shoulder external rotation exercises ranging from 0°–90°at a constant rhythm of 40 bpm using a metronome. Notably, the 0°–90° external rotation and 90°–0° external rotation were performed at the same speed.

**Figure 4:**
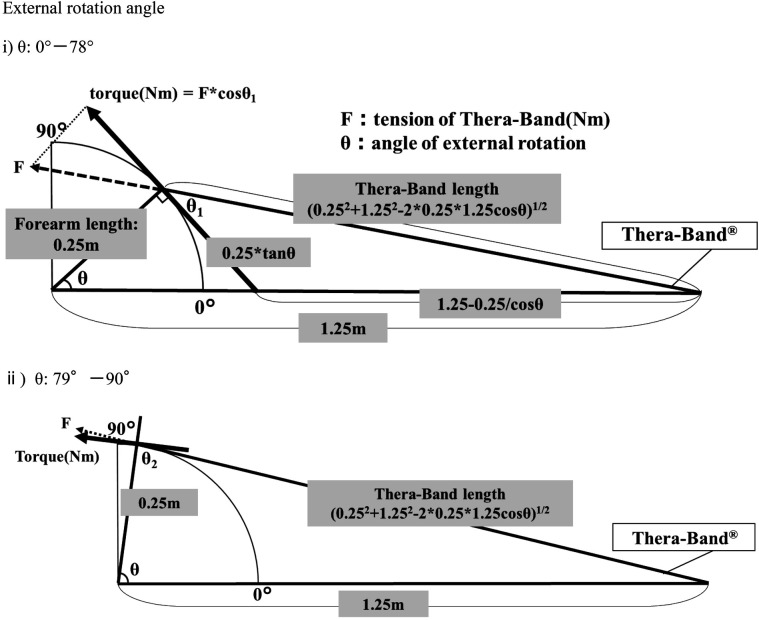
Calculation of workload during shoulder joint external rotation exercises. The direction of tension of Thera-Band and shoulder joint external rotation torque coincided at 78°.

We adjusted the repetition of the exercise to ensure that the total workload across the conditions was equal. Specifically, the number of repetitions was set to 20 for the low-load condition, 11 for the medium-load condition, and 6 for the high-load condition. Furthermore, the forearm length was set to 0.25 m in this study based on the mean forearm length of healthy adult males shown in previous studies (16, 17). The length of Thera-Band® at the beginning of the exercise was 1 m, hence the cosine theorem was applied to the triangle made by the sum of the lengths of Thera-Band® and forearm at the beginning of the exercise (1.25 m), forearm (0.25 m), and Thera-Band® during shoulder external rotation exercise. The cosine theorem was used to calculate the length of the Thera-Band® for each degree of external rotation (Thera-Band length) (Figure 4). The results showed that the Thera-Band® lengthened from 1–1.28 m during movement from 0°–90° of shoulder external rotation. Next, the Thera-Band® was fixed to the shoulder joint attachment of Biodex System 4, with the arm length adjusted to 0.25 m to determine the torque required to pull the Thera-Band® (Thera-Band torque). The Thera-Band® torque was measured by extending the arm in 3 cm increments from 1 m. The tension of a 50 cm Thera-Band stretched to 1 m, measured using BIODEX, was calculated by subtracting the arm length of 0.25 m from the recorded values. The tensions were 16.6 N for the yellow band, 30.3 N for the black band, and 54.2 N for the silver band. Using Hooke's law, we calculated an approximate formula with the amount of displacement of Thera-Band® on the horizontal axis and the Thera-Band® torque obtained earlier on the vertical axis. The Thera-Band® torque per degree of shoulder external rotation from 0°–90° was calculated using the values of the approximate formula obtained. Notably, the vector of the Thera-Band® torque and the shoulder external rotation torque coincide between 78° and 79°, hence the following equations were used to calculate the Thera-Band® torque for each degree of external rotation from 0°–78° and from 79°–90°. The workload from 0°–90° was calculated using the following equation, and the value was multiplied by two to calculate the workload required to make one round trip from 0°–90°. The results showed that the workload per exercise for the low-, medium-, and high-load conditions were 10.28 J, 18.59 J, and 34.47 J, respectively. It is recommended that rotator cuff exercises be performed at a low-load for approximately 20 repetitions per set (9); therefore, the number of repetitions was set to be equivalent to a total workload of approximately 205 J, which is the total workload of 20 at low-load. This resulted in 11 repetitions at medium load and 6 at high load.

Shoulder external rotation from 0°–78°.

$$\begin{array}{rl} & \sum \;\{(\mathrm{Thera}-\mathrm{Band}\;\mathrm{torque})\;\times \;\cos\;\theta 1\;\times \;0.017\}\;\\ & \cos\;\theta 1=\{(0.25\;\times \;\tan\;\theta {)}^{2}+\;(\mathrm{Thera}-\mathrm{Band}\;\mathrm{length}{)}^{2}\\ & \;-\;\;{\left(1.25-\frac{0.25}{\begin{array}{c} \cos\;\theta \end{array}}\right)}^{2}÷\{2\;\times \;(0.25\;\times \;\tan\;\theta )\\ & \;\times \;(\mathrm{Thera}-\mathrm{Band}\;\mathrm{length})\} \end{array}$$

Shoulder external rotation from 79°–90°.

$$\begin{array}{rl} & \sum \;\{(\mathrm{Thera}-\mathrm{Band}\;\mathrm{torque})\;\times \;\sin\;\theta 2\;\times \;0.017\}\\ & \cos\;\theta 2=\{0.25^{2}+\;{\left(\mathrm{Thera}-\mathrm{Band}\;\mathrm{length}\right)}^{2}-1.25^{2}\}\;\\ & \;\;\;÷\{2\times 0.25\;\times \;(\mathrm{Thera}-\mathrm{Band}\;\mathrm{length})\} \end{array}$$

0.017: 1 radian.
*θ*: Angle of shoulder external rotation.

### 2.5 Electromyography amplitude, torque, and frequency

The participants performed concentric contraction shoulder external rotation tasks on the Biodex System 4 before and after the exercises. During these tasks, shoulder external rotation torque and EMG amplitude of the infraspinatus, teres minor, and posterior deltoid muscles were measured. The task was performed three times at an angular velocity of 60°/second in the range of 0°–90°of the shoulder rotation. During the measurements, the participant was seated with the shoulder joint positioned at 90° abduction on the Biodex, and the upper body immobilized using the non-stretchable straps provided on the Biodex during the Thera-Band® exercises. The position was set so that the center of rotation of the shoulder joint coincided with the dynamometer.

### 2.6 Data processing

The torque and angle signals obtained by synchronizing the Biodex System 4 to the Ultium EMG wireless myoelectric system were used to analyze the shoulder external rotation torque before and after the shoulder external rotation exercise. The maximum value of the torque at 0°–90° of external rotation was calculated, and the highest value among the three trials was considered as the peak torque based on the angle signal. Furthermore, in exercises using rubber bands, the tension increases as the external rotation angle increases due to the characteristic of rubber, which shows increasing tension with greater displacement. Therefore, the workload was calculated to understand the ability of exerting torque throughout the entire exercise. The value obtained from the angle signal was converted to radians, the product of radians and torque was summed from 0°–90° of shoulder external rotation, and the highest of the three was used in the analysis as the maximum work capacity. For peak torque and maximum workload, the relative values with respect to the pre-exercise (Pre) values were calculated using the following equations.

Relativevaluesforpeaktorqueandmaximumworkload={afterexercise−Pre}÷Pre×100

EMG amplitude data before and after shoulder external rotation exercises were full-wave rectified using a digital filter (Noraxon) and subsequently smoothed in a 100 ms window. The values were normalized [percentage (%) MVIC] for analysis as muscle activity using the mean of the mid 3 s of a 5 s MVIC test. The %MVIC was used as the amount of EMG amplitude in the analysis. Furthermore, the muscle activity ratio was calculated by dividing the EMG amplitude (%MVIC) of each muscle by the sum of the EMG amplitude of the cranial part and caudal part of the infraspinatus, teres minor, and posterior deltoid. Because of the characteristics of the elastic bands, the effects of the exercise could differ depending on the angle of shoulder external rotation, hence, the exercise range was divided into 0°–30°, 31°–60°, and 61°–90° for analysis. For the EMG amplitude and muscle activity ratio for each muscle, the average for each exercise range was calculated, and the peak torque for each exercise range was calculated and analyzed for the shoulder external rotation torque. The average frequency during concentric contraction shoulder external rotation before and after the exercise was also analyzed because fatigue from the exercise may have affected the results.

In shoulder external rotation exercises using the Thera-Band®, the longer the distance the Thera-Band® is pulled, the greater the tension. Thus, the intensity of the exercise increases as the external rotation angle increases. The shoulder external rotation torque when Thera-Band® was pulled for each 10° of external rotation, was divided by the peak torque during the MVIC test for each participant to calculate the intensity during the exercise (%) (Table 1). For EMG amplitude during the exercise, the average of the first five EMG amplitudes for each exercise condition was calculated, and normalized to the value during the MVIC test. The relative value was calculated as one using the low-load condition (Table 2).

**Table 1:** Intensities during exercise.

Angle (°)	Low (%)	Medium (%)	High (%)
10	3.16 ± 0.67	5.73 ± 1.29	9.92 ± 2.29
20	6.22 ± 1.33	11.27 ± 2.54	19.59 ± 4.51
30	9.10 ± 1.94	16.45 ± 3.70	28.77 ± 6.63
40	11.70 ± 2.50	21.09 ± 4.75	37.19 ± 8.57
50	13.94 ± 2.98	25.08 ± 5.65	44.61 ± 10.28
60	15.75 ± 3.36	28.27 ± 6.37	50.77 ± 11.70
70	17.09 ± 3.65	30.60 ± 6.89	55.45 ± 12.78
80	17.92 ± 3.83	32.00 ± 7.21	58.49 ± 13.48
90	18.54 ± 3.96	33.04 ± 7.44	60.88 ± 14.03

Mean ± SD.

The value was calculated by dividing the external rotation torque of the shoulder joint required to pull the Thera-Band by the maximum muscle force during the maximum isometric voluntary contraction.

**Table 2:** Muscle activity during exercises with elastic bands.

	Low	Medium	High	*p* value
Infraspinatus				
Cranial Part	1	1.48 ± 0.36	2.40 ± 0.77	<0.001
Caudal Part	1	1.38 ± 0.51	2.02 ± 0.54	<0.001
Teres Minor	1	1.43 ± 0.27	2.52 ± 0.69	<0.001
Posterior Deltoid	1	1.29 ± 0.34	2.29 ± 0.58	<0.001

The average of the first five cycles of low, medium, and high-intensity exercises was calculated, and the relative activity was calculated with the low-intensity set as 1.

1-way repeated-measures analysis of variance (ANOVA) (parametric) or the Kruskal–Wallis test (nonparametric) was performed.

### 2.7 Statistical processing

We performed a repeated-measure two-way analysis of variance (ANOVA) for load (low-, medium-, and high-load) × time (Pre, 10 min, 20 min, and 30 min) on peak shoulder external rotation torque and maximal workload across the entire range of motion before and after exercise. Furthermore, a repeated-measures 2-way ANOVA was performed for exercise range (0°–30°, 31°–60°, 61°–90°) × time for shoulder external rotation torque, the muscle activity ratio, and EMG amplitude of each muscle. Normality tests were conducted using the Shapiro–Wilk test, and for the variables that did not follow a normal distribution, the Wilcoxon signed-rank test was performed instead. Statistical analysis was performed using the Statistical Package for Social Sciences version 29 (IBM) and the significance level was set at 5%.

## 3 Results

### 3.1 Change in peak torque and maximal workload

There was no interaction (*p* = 0.168) between load (low-, medium-, high-load) × time (Pre, 10 min, 20 min, 30 min) for peak torque during 0°–90° shoulder external rotation. However, a main effect of time was observed, with the peak torque increasing significantly from Pre to 10 min. Regarding maximal workload, there was an interaction between load × time (*p* = 0.033) and a main effect of time (*p* < 0.001). Furthermore, *post-hoc* test showed a significant increase in peak torque at 10 min with low-load (7.68 ± 8.87%: *p* < 0.001) and high-load (8.92 ± 10.29%: *p* < 0.001) compared with Pre. At 20 min after exercise, the peak torque of medium-load condition (4.92 ± 5.31%) increased compared with Pre (*p* = 0.01). Post-activity potentiation (18), characterized by an increase in exerted torque lasting up to 10–15 min after submaximal muscle contraction without fatigue, is believed to include effects due to phosphorylation of myosin light chains rather than changes in muscle activity (19). Therefore, the change at 10 min was likely due to post-activation potentiation effect. Furthermore, 30 min after exercise, there was no difference from Pre in either peak torque or maximal workload. Therefore, we focused on the changes at 20 min from Pre under low-, medium-, and high-load conditions. The results showed that there were no significant differences in shoulder external rotation torque between Pre and 20 min under the low- and high-load conditions. However, at medium-load condition, there were significant differences between 0°–30° (Pre: 25.35 ± 5.43 Nm, 20 min: 25.96 ± 5.24 Nm: *p* = 0.027), 31°–60° (Pre: 24.28 ± 5.52 Nm, 20 min: 25.42 ± 5.45 Nm: *p* = 0.003), 61°–90° (Pre: 23.65 ± 5.41 Nm, 20 min: 24.58 ± 5.95 Nm: *p* = 0.007), with the torque increasing from Pre to 20 min.

### 3.2 Muscle activity ratio

**Figure 5:**
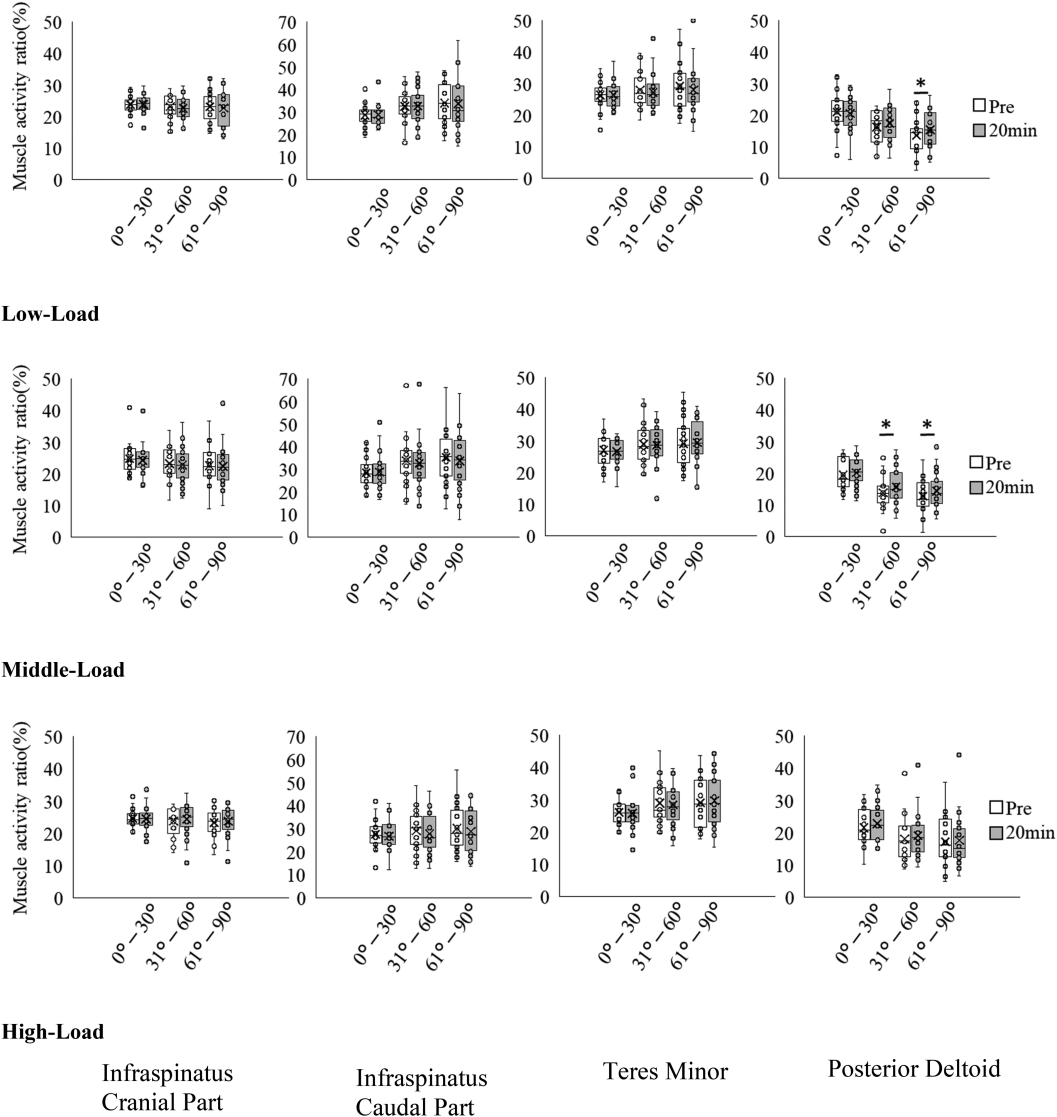
Changes in muscle activity ratio for each muscle before and after exercise. *p < 0.05.

Results for muscle activity ratio before and 20 min after exercise are shown in Table 3 and Figure 5. In the cranial part and caudal part of the infraspinatus and teres minor, there was no significant differences before and 20 min after exercise in the low-, medium-, and high-load conditions. In the posterior deltoid, at low-load, an interaction was observed between exercise range × time (*p* = 0.044), with an increase in the muscle activity ratio from 61°–90° at 20 min (15.40 ± 6.03%) compared with Pre (13.59 ± 5.70%). At medium-load, there was no exercise range × time interaction (*p* = 0.370); however, increase in the muscle activity ratio increased from 31°–60° of shoulder external rotation (Pre: 13.80 ± 5.05%, 20 min: 15.78 ± 5.37%: *p* = 0.023) and 61°–90° (Pre: 12.74 ± 5.44%, 20 min: 14.35 ± 5.55%: *p* = 0.047), increased from Pre to 20 min, indicating a main effect of time. At high-load, there was no significant differences before and 20 min after exercise.

**Table 3:** Changes in muscle activity ratio for each muscle before and after exercise.

		Infraspinatus		Teres minor (%)	Posterior deltoid (%)
		Cranial part (%)	Caudal part (%)		
Low					
0°–30°	Pre	23.8 ± 2.9	28.8 ± 4.6	26.4 ± 4.3	21.0 ± 6.0
	20 min	24.0 ± 3.2	28.9 ± 5.7	26.5 ± 4.0	20.7 ± 5.5
31°–60°	Pre	23.2 ± 4.1	32.5 ± 7.1	28.0 ± 5.1	16.3 ± 5.5
	20 min	22.6 ± 4.0	32.7 ± 8.1	27.3 ± 5.6	17.4 ± 5.5
61°–90°	Pre	23.2 ± 4.8	34.0 ± 8.8	29.3 ± 7.1	13.6 ± 5.7
	20 min	22.8 ± 5.4	33.8 ± 11.4	28.1 ± 7.3	15.4 ± 6.0*
Interaction effect	*p* value	0.581		0.175	0.044
Time	*p* value	0.551		0.277	0.185
Angle	*p* value	0.254		0.033	<0.001
Statistical Method		RM-2wayANOVA	Wilcoxon	RM-2wayANOVA	RM-2wayANOVA
Medium					
0°–30°	Pre	24.9 ± 4.8	28.6 ± 6.6	27.1 ± 5.1	19.4 ± 4.7
	20 min	24.3 ± 5.0	28.9 ± 7.6	26.6 ± 3.9	20.2 ± 4.2
31°–60°	Pre	23.2 ± 5.0	34.0 ± 10.4	29.0 ± 6.2	13.8 ± 5.1
	20 min	22.9 ± 5.6	32.7 ± 11.4	28.6 ± 6.2	15.8 ± 5.4*
61°–90°	Pre	22.7 ± 5.9	35.3 ± 11.6	29.3 ± 7.3	12.7 ± 5.4
	20 min	22.4 ± 6.6	33.7 ± 12.6	29.6 ± 6.9	14.4 ± 5.6*
Interaction effect	*p* value			0.497	0.370
Time	*p* value			0.837	0.023
Angle	*p* value			0.011	<0.001
Statistical Method		Wilcoxon	Wilcoxon	RM-2wayANOVA	RM-2wayANOVA
High					
0°–30°	Pre	24.6 ± 3.1	27.6 ± 6.2	26.2 ± 3.8	21.6 ± 5.6
	20 min	24.5 ± 3.9	26.8 ± 6.1	25.9 ± 5.4	22.8 ± 5.8
31°–60°	Pre	23.8 ± 4.3	29.2 ± 8.6	29.0 ± 6.5	18.1 ± 7.1
	20 min	24.5 ± 5.0	27.9 ± 8.4	28.5 ± 6.4	19.2 ± 6.7
61°–90°	Pre	23.4 ± 4.2	30.2 ± 9.9	29.1 ± 7.7	17.3 ± 7.5
	20 min	23.8 ± 4.6	28.8 ± 9.5	29.7 ± 8.3	17.6 ± 8.0
Interaction effect	*p* value	0.511	0.775	0.446	
Time	*p* value	0.377	0.011	0.934	
Angle	*p* value	0.327	0.282	0.007	
Statistical Method		RM-2wayANOVA	RM-2wayANOVA	RM-2wayANOVA	Wilcoxon

Mean ± SD; Pre, pre-exercise.

* *p* < 0.05 compared to Pre.

### 3.3 Electromyography amplitude of each muscle

**Figure 6:**
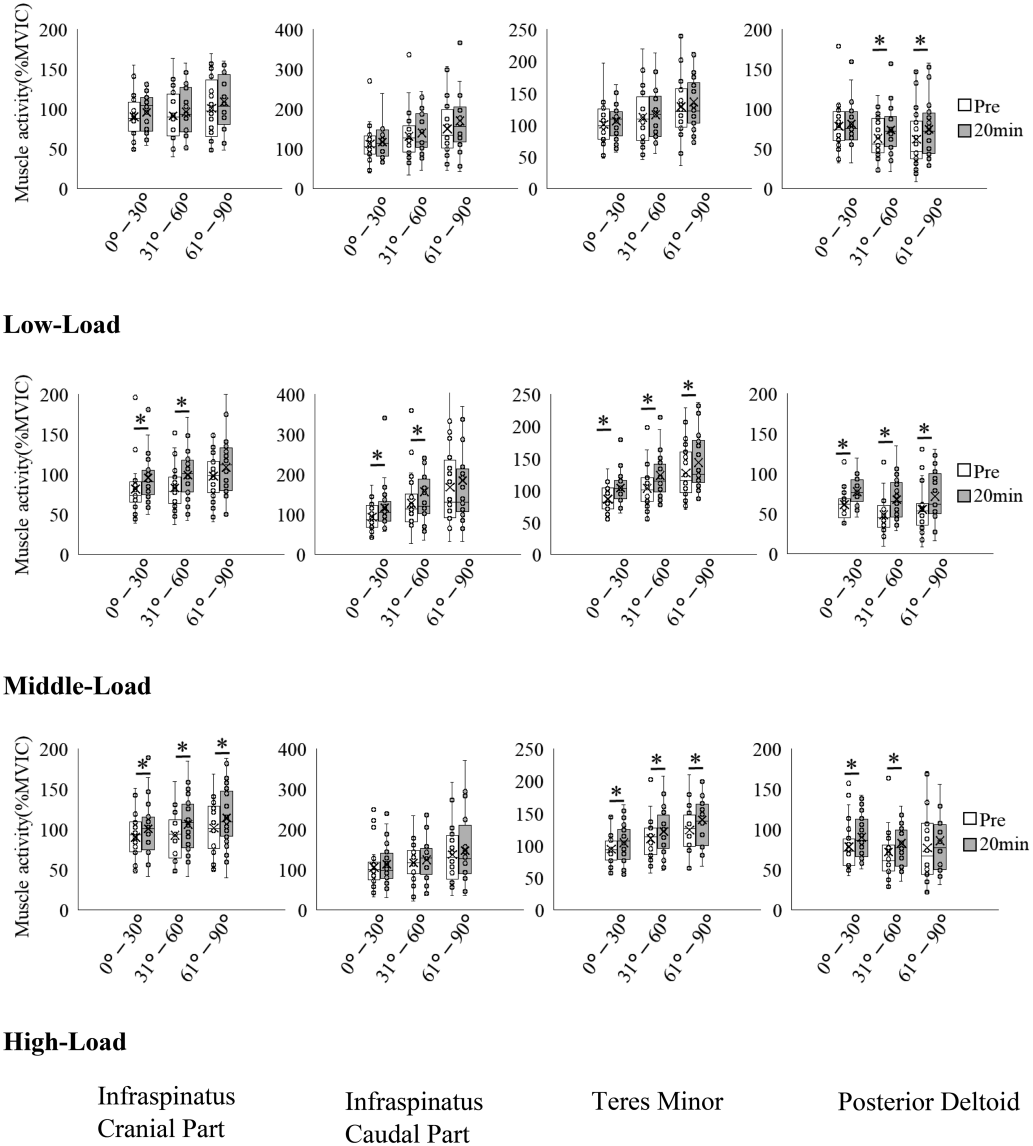
Changes in the muscle activity before and after exercise. *p < 0.05.

Results of EMG amplitude before and 20 min after exercise are shown in Table 4 and Figure 6, and further information is available in Table 5. At low-load, no significant differences were observed between Pre and 20 min in the cranial part and caudal part of the infraspinatus and teres minor. However, there was an increase in EMG amplitude from Pre to 20 min in the posterior deltoid, with significant differences observed at 31°–60° (Pre: 63.43 ± 27.36% MVIC, 20 min: 73.87 ± 30.61% MVIC, *p* = 0.034) and at 61°–90° (Pre: 62.10 ± 39.00% MVIC, 20 min: 74.70 ± 37.76% MVIC, *p* = 0.002). At medium- and high-loads, EMG amplitude increased from Pre to 20 min in the infraspinatus, teres minor, and posterior deltoid, respectively.

**Table 4:** Changes in the muscle activity before and after exercise.

		Infraspinatus		Teres minor (% MVIC)	Posterior deltoid (% MVIC)
		Cranial part (% MVIC)	Caudal part (% MVIC)		
Low					
0°–30°	Pre	90.7 ± 27.3	112.0 ± 45.5	100.8 ± 33.7	78.5 ± 30.2
	20 min	96.1 ± 25.3	117.0 ± 40.1	106.2 ± 28.2	81.6 ± 28.3
31°–60°	Pre	91.7 ± 32.8	132.0 ± 62.8	111.9 ± 43.4	63.4 ± 27.4
	20 min	96.4 ± 31.6	140.4 ± 53.9	116.3 ± 40.6	73.9 ± 30.6*
61°–90°	Pre	101.1 ± 37.7	150.9 ± 68.5	129.0 ± 51.6	62.1 ± 39.0
	20 min	109.3 ± 34.7	171.1 ± 90.7	136.4 ± 55.8	74.7 ± 37.8*
Interaction effect	*p* value	0.771			
Time	*p* value	0.099			
Angle	*p* value	0.007			
Statistical Method		RM-2wayANOVA	Wilcoxon	Wilcoxon	Wilcoxon
Medium					
0°–30°	Pre	82.2 ± 33.3	93.7 ± 34.7	86.4 ± 21.7	61.8 ± 18.0
	20 min	96.0 ± 29.0*	117.7 ± 58.3*	104.3 ± 25.0*	78.0±16.9*
31°–60°	Pre	83.4 ± 27.4	128.4 ± 70.5	103.8 ± 32.9	49.0 ± 22.5
	20 min	99.3 ± 30.4*	157.2 ± 120.3*	123.9 ± 39.2*	68.1 ± 26.1*
61°–90°	Pre	98.0 ± 29.6	168.1 ± 98.9	127.6 ± 42.6	55.6 ± 28.9
	20 min	109.3 ± 36.9	184.9 ± 148.2	143.8 ± 44.3*	71.6 ± 31.6*
Interaction effect	*p* value	0.783			
Time	*p* value	<0.001			
Angle	*p* value	0.006			
Statistical Method		Wilcoxon	Wilcoxon	Wilcoxon	RM-2wayANOVA
High					
0°–30°	Pre	90.4 ± 27.7	106.6 ± 53.6	94.6 ± 25.3	78.5 ± 30.5
	20 min	101.0 ± 34.8*	113.8 ± 53.0	104.5 ± 31.0*	90.7 ± 26.8*
31°–60°	Pre	93.1 ± 31.0	117.1 ± 47.7	110.9 ± 32.1	72.2 ± 42.9
	20 min	106.5 ± 35.1*	124.9 ± 54.1	122.4 ± 36.4*	83.2 ± 38.0*
61°–90°	Pre	102.1 ± 31.5	137.9 ± 69.7	124.1 ± 35.9	77.3 ± 43.1
	20 min	114.8 ± 37.7*	148.3 ± 84.9	140.1 ± 48.2*	86.2 ± 53.4
Interaction effect	*p* value	0.765	0.824	0.447	
Time	*p* value	0.002	0.075	0.010	
Angle	*p* value	0.008	0.004	<0.001	
Statistical Method		RM-2wayANOVA	RM-2wayANOVA	RM-2wayANOVA	Wilcoxon

%MVIC, percentage of maximum voluntary isometric contraction; Pre, pre-exercies.

* *p* < 0.05 compared to Pre.; Mean ± SD.

**Table 5:** Raw EMG data: changes in the muscle activity before and after exercise.

		Infraspinatus		Teres minor (*μ*V)	Posterior deltoid (*μ*V)
		Cranial part (*μ*V)	Caudal part (*μ*V)		
Low					
0°–30°	Pre	342.5 ± 145.5	441.0 ± 291.3	303.3 ± 95.7	394.5 ± 167.4
	20 min	358.2 ± 116.8	452.6 ± 200.2	328.4 ± 113.3	418.3 ± 187.6
31°–60°	Pre	336.8 ± 129.4	508.0 ± 330.2	329.3 ± 99.6	330.8 ± 192.7
	20 min	351.8 ± 109.3	526.7 ± 232.5	347.7 ± 104.0	394.6 ± 226.2
61°–90°	Pre	374.5 ± 150.8	558.9 ± 253.0	376.8 ± 115.5	311.1 ± 206.4
	20 min	399.1 ± 122.0	614.1 ± 273.0	403.3 ± 119.8	392.4 ± 254.2
Medium					
0°–30°	Pre	314.0 ± 130.4	377.5 ± 156.6	280.1 ± 100.1	361.0 ± 161.1
	20 min	374.6 ± 148.6	490.3 ± 256.1	336.1 ± 111.0	470.7 ± 228.0
31°–60°	Pre	320.2 ± 122.5	491.3 ± 211.1	333.4 ± 119.7	289.6 ± 169.6
	20 min	381.1 ± 142.4	618.5 ± 407.8	392.8 ± 122.9	427.9 ± 277.8
61°–90°	Pre	375.7 ± 138.2	629.6 ± 301.3	408.2 ± 153.8	320.5 ± 196.0
	20 min	424.7 ± 180.2	695.2 ± 448.6	455.2 ± 135.4	454.1 ± 334.6
High					
0°–30°	Pre	334.6 ± 125.9	463.5 ± 190.2	303.5 ± 77.6	444.7 ± 182.2
	20 min	370.2 ± 146.7	504.2 ± 225.5	332.8 ± 95.9	527.7 ± 224.8
31°–60°	Pre	328.9 ± 78.2	523.8 ± 240.5	351.8 ± 88.2	405.3 ± 208.1
	20 min	380.2 ± 109.1	574.0 ± 349.2	387.1 ± 100.3	489.2 ± 250.3
61°–90°	Pre	368.7 ± 104.2	606.3 ± 286.4	392.0 ± 96.7	447.0 ± 270.9
	20 min	415.9 ± 138.5	646.0 ± 413.6	440.6 ± 125.7	506.7 ± 315.4

Mean ± SD; Pre, pre-exercise.

### 3.4 Average frequency

The results of the average frequencies before and 20 min after the exercise are presented in Table 6. No significant decreases were observed in any of the cranial or caudal parts of the infraspinatus, the teres minor, or the posterior deltoid from Pre to 20 min under low-, medium-, or high-load conditions, based on the Wilcoxon signed-rank test.

**Table 6:** Changes in the frequency before and after exercise.

		Infraspinatus		Teres minor (Hz)	Posterior deltoid (Hz)
		Cranial part (Hz)	Caudal part (Hz)		
Low	Pre	61.19 ± 22.02	56.07 ± 24.39	55.35 ± 20.82	56.60 ± 25.66
	20 min	61.90 ± 26.82	56.70 ± 29.50	56.24 ± 26.53	59.75 ± 30.11
	*p* value	0.9320.607	0.797		0.004
Medium	Pre	63.45 ± 26.05	58.77 ± 27.07	58.08 ± 25.17	58.51 ± 30.42
	20 min	63.56 ± 28.40	58.46 ± 28.03	58.34 ± 28.34	60.80 ± 30.08
	*p* value	0.954	0.668	0.864	<0.001
High	Pre	68.16 ± 32.11	60.08 ± 29.24	59.40 ± 25.21	60.10 ± 29.31
	20 min	64.29 ± 24.29	57.43 ± 25.30	58.08 ± 25.30	59.41 ± 27.41
	*p* value	0.732	0.278	0.354	0.067

Mean ± SD; Pre, pre-exercise.

The Wilcoxon signed-rank test was used to analyze the differences between conditions, as the data did not follow a normal distribution according to the Shapiro–Wilk test.

## 4 Discussion

In this study, we aimed to determine the effects of shoulder external rotation exercises at different loads on external rotation torque and changes in the EMG amplitude and muscle activity ratio of the infraspinatus, teres minor, and deltoid, by using three types of Thera-Band® with different tensions. Results showed that after 20 min of exercise, the muscle activity ratio in the posterior deltoid muscle increased only in the range of 61°–90° of shoulder external rotation in the low-load condition, whereas, in the medium-load condition it increased regardless of the angle of external rotation. In contrast, there was no change in the muscle activity ratio in the high-load condition. Moreover, the EMG amplitude of all muscles increased regardless of external rotation angle in the medium- and high-load conditions; however, shoulder external rotation torque increased only in the medium-load condition.

We hypothesized that increasing the load of shoulder external rotation exercises would not change the muscle activity ratio in the shoulder external rotators after the exercise. However, the muscle activity ratio did change before and after the exercises under low-and medium-load conditions. Notably, the muscle activity ratio in the posterior deltoid increased under low- and medium-load conditions. Conversely, the muscle activity ratio in the infraspinatus and teres minor decreased; however, each muscle did not significantly differ before or after the exercises. In this study, the repetition of exercises differed among conditions to unify the total workload of the exercises: 20 repetitions in the low-load condition, 11 repetitions in the medium-load condition, and 6 repetitions in the high-load condition. Additionally, the exercises in all conditions were performed at the same tempo; however, the time required for the exercises in the high-load condition was 1/3 of that in the low-load condition. Therefore, it is likely that the shoulder external rotation exercise in the high-load condition with fewer repetitions were not enough to change the muscle activity ratio in the posterior deltoid. Furthermore, the muscle activity ratio in the posterior deltoid did not change in the low-load condition from 0°–60° of external rotation. Notably, the shoulder external rotation torque required to pull the Thera-Band® in the low-load condition was 4.5 Nm at 61° of shoulder external rotation; however, it was 8.2 Nm at the same angle for medium-load. In the low-load condition, the tension of the Thera-Band® was not strong enough to increase the muscle activity ratio of the posterior deltoid from 0°–60° of shoulder external rotation.

The EMG amplitude increased only in the posterior deltoid in the low-load condition, whereas it increased in all muscles after the exercise in the medium- and high-load condition. After the exercises performed in this study, the EMG amplitude of the infraspinatus and teres minor did not increase in the low-load condition, whereas this amplitude in all muscles increased in the medium- and high-load conditions. Previous studies examining EMG amplitude during shoulder external rotation exercises have reported increases in both infraspinatus and posterior deltoid EMG amplitude with increasing exercise load for both exercises in the 90° shoulder abduction position (20) and in the 0° shoulder abducted position (7, 21). In the present study, the EMG amplitude of the cranial part and caudal part of the infraspinatus, teres minor, and the posterior deltoid during the exercises was greater at high-, medium-, and low-loads, in the same order as in the previous studies. It was also reported that shoulder external rotation exercises in the 90° shoulder abducted position resulted in greater EMG amplitude in the infraspinatus and posterior deltoid compared with the 0° shoulder abducted position (13, 22). In addition, the infraspinatus/posterior deltoid muscle activation ratio was greater during low-load exercises than during high-load exercises (6). Therefore, it is likely that performing the exercise in the 90° shoulder abduction position increased the EMG amplitude in the shoulder external rotation muscle group and increased the muscle activity ratio in the deltoid during the exercise, which also led to an increase in the EMG amplitude after the exercise. We believe that the medium-load condition of this study, performed at 90° shoulder abduction, is effective in increasing short-term shoulder external rotation torque based on these results. On the other hand, the increased activation of the posterior deltoid muscle during shoulder joint exercises may generate a shearing force on the humeral head. Repeated daily performance of shoulder external rotation exercises could potentially lead to shoulder joint pain, as this force may displace the humeral head from its centered position in the glenoid fossa. Future studies are needed to assess the risk of developing shoulder joint pain with continuous practice of these exercises and to explore optimal exercise protocols that minimize this risk.

The high-load condition did not increase shoulder external rotation torque, despite increased EMG amplitude in all muscles. Exercises with Thera-Band® in this study were performed in the 90° shoulder abducted position; however, the entire arm was not fixed. Therefore, compensations such as scapular elevation, shoulder abduction or horizontal extension may have appeared. The uncontrolled compensatory movements of the shoulder joint during the exercises may have resulted in increased EMG amplitude other than the external rotator muscles. A previous study showed that the shoulder external rotators and the anterior and middle deltoid, supraspinatus, upper trapezius, and serratus anterior were more active in the 90° shoulder abducted position than in the 0° abducted position (22). Our study did not record EMG amplitude other than the shoulder external rotator muscles. It is possible that muscles other than the shoulder external rotators also worked during the high-load exercise, resulting in an increase in EMG amplitude after the exercise. Furthermore, the posterior deltoid muscle, whose EMG amplitude was measured in this study, also has abduction and horizontal extension actions along with shoulder external rotation. It is possible that the posterior deltoid was involved in the compensatory movements of shoulder abduction and horizontal extension that may have emerged during the high-load condition exercise. Based on the above, we believe that the high-load condition in this study did not lead to an increase in shoulder external rotation torque despite the increase in the EMG amplitude of the shoulder external rotators. We believe that the fatigue of exercise is not a factor resulting in the lack of increase in shoulder external rotation torque for the following reasons. The average frequency of concentric shoulder external rotation did not change after the exercise, the post-exercise measurements were taken 20 min after the exercise and the number of exercises in the high-load condition was only six.

Although peak torque did not change from pre-exercise, maximal workload significantly increased at 20 min post-exercise under medium-load condition. Additionally, there was no significant difference in external rotation torque between pre and 20 min after exercise within the range of 0°–30°; however, significant increases were observed in the range of 31°–60° and 61°–90°. The increase in shoulder external rotation torque beyond 31° is likely due to the properties of the Thera-Band®, as the resistance increases with increasing angle of external rotation. On the other hand, peak torque in shoulder external rotation is reported to occur in the early range of external rotation (11). The fact that the exercise tasks in this study did not lead to an increase in peak torque may be due to the relatively low workload imposed by the Thera-Band® in the early range of external rotation, where peak torque is typically exerted. Furthermore, the increase in maximal workload is considered to be due to the increase in external rotation torque within the larger external rotation ranges where Thera-Band® resistance increased in the latter half of the rotation.

### 4.1 Limitation

In this study, the elbow position was not fixed during the exercises with Thera-Band®, which may have resulted in the appearance of compensatory movements; however, no evaluation was made regarding compensatory movements. In addition, the EMG amplitude of muscles other than that of the shoulder external rotators was not recorded, so the influence of EMG amplitude other than those on the results remains unclear. Therefore, it is necessary to record the EMG amplitude other than the shoulder external rotators and examine the influence of muscles in this context. In addition, this study used the same type of Thera-Band® in all participants. The relative exercise intensity differed from one participant to the other due to differences in muscle strength among the participants. In this study, we used an elastics tool, Thera-Band®, in order to examine the method commonly used in the field. However, we believe that further investigation using equipment such as dumbbells is needed so that we can fine-tune the exercise load.

## 5 Conclusion

The changes in torque, EMG amplitude, and the muscle activity ratio of each muscle after shoulder external rotation exercises were not uniform depending on the load of the exercises. Therefore, it is necessary to use different exercise loads based on the purpose of the exercise.

## Data Availability

The original contributions presented in the study are included in the article/Supplementary Material, further inquiries can be directed to the corresponding author.
